# Dissection of structural dynamics of chromatin fibers by single-molecule magnetic tweezers

**DOI:** 10.1007/s41048-018-0064-0

**Published:** 2018-08-29

**Authors:** Xue Xiao, Liping Dong, Yi-Zhou Wang, Peng-Ye Wang, Ming Li, Guohong Li, Ping Chen, Wei Li

**Affiliations:** 1 National Laboratory for Condensed Matter Physics and Key Laboratory of Soft Matter Physics, Institute of Physics, Chinese Academy of Sciences, Beijing, 100190 China; 2 National Laboratory of Biomacromolecules, CAS Center for Excellence in Biomacromolecules, Institute of Biophysics, Chinese Academy of Sciences, Beijing, 100101 China; 3 University of Chinese Academy of Sciences, Beijing, 100049 China

**Keywords:** Magnetic tweezers, Chromatin fiber, Dynamics, Single molecule

## Abstract

The accessibility of genomic DNA, as a key determinant of gene-related processes, is dependent on the packing density and structural dynamics of chromatin fiber. However, due to the highly dynamic and heterogeneous properties of chromatin fiber, it is technically challenging to study these properties of chromatin. Here, we report a strategy for dissecting the dynamics of chromatin fibers based on single-molecule magnetic tweezers. Using magnetic tweezers, we can manipulate the chromatin fiber and trace its extension during the folding and unfolding process under tension to investigate the dynamic structural transitions at single-molecule level. The highly accurate and reliable *in vitro* single-molecule strategy provides a new research platform to dissect the structural dynamics of chromatin fiber and its regulation by different epigenetic factors during gene expression.

## Introduction

In eukaryotic cells, genomic DNA is wrapped on histones to form the nucleosome (Richmond and Davey 2003), which is the basic repeating unit of chromatin, and then further folds to condensed chromatin fibers (Bickmore and van Steensel 2013). But the transcription and replication of DNA require the accessibility of DNA double-helix structure by transcription factors and polymerases. The packing density and structural dynamics of chromatin fibers are regulated by many epigenetic factors, including chromatin modifications, histone chaperones, histone variants, chromatin remodelers, and chromatin architectural proteins. For the genomic regions where gene expression are active, a series of epigenetic modifications (Zentner and Henikoff 2013), enzymes (Maier *et al*. 2000), and transcription factors (Li *et al*. 2016b; Pavri *et al*. 2006) assist in unfolding of chromatin fibers to expose DNA, allowing the transcription and replication to proceed. When the transcription or duplication is completed, the chromatin fiber needs to be reassembled (Fleming *et al*. 2008). Therefore, deciphering the structural dynamics and its epigenetic regulation of chromatin fibers is the basis for understanding the epigenetic regulation of gene-related biological processes including transcription and DNA replication.

Traditional biochemical methods have shown some difficulty in studying the dynamic structural changes of chromatin fibers (Fleming *et al*. 2008; Georgel *et al*. 2003). For example, the properties of chromatin fibers obtained in gel electrophoresis and circular dichroism are the sum of a huge number of molecules involved in the reaction; thus it is impossible to characterize the dynamic process of structural changes of chromatin fibers. Although high-resolution imaging techniques, such as X-ray and Cryo-electron microscopy (Cryo-EM), have successfully determined the high-resolution structures of the nucleosome (Luger *et al*. 1997) and the 30-nm chromatin fiber (Song *et al*. 2014), these techniques are only suitable for studying static structures and cannot monitor the dynamic structural transitions of the nucleosome and chromatin fibers. Fluorescence resonance transfer (FRET) technique can track the dynamic changes of chromatin structure at single-molecule level (Chen *et al*. 2016; Fierz *et al*. 2011; Ngo and Ha 2015). However, in most time, fluorescence probe quenches in a few minutes; thus it is difficult to monitor the structural transitions in such a short-time window.

Single-molecule force spectroscopy is an ideal technique to study the structural change of biological macromolecules, especially the nucleosome and chromatin fibers. By applying different external forces on the single chromatin fiber or mononucleosome, we can manipulate conformational changes of the molecule, which could be monitored by tracking the extension of the chromatin fiber or mononucleosome. We can also deduce the stability and assembly pattern of the chromatin fiber or mononucleosome by analyzing the step size and the transition force of structural intermediates. The main techniques of single-molecular force spectroscopy include atomic force microscopy (AFM; Piontek and Roos 2018), optical tweezers (Neuman and Block 2004), and magnetic tweezers (Gosse and Croquette 2002). AFM captures biological macromolecules by a chemically modified probe and applies a pulling force, but the pulling force is usually very large and it is difficult to ensure only one single-molecule is captured. The optical tweezers require the two ends of molecule binding to two polystyrene beads respectively, and then the optical tweezers grab the polystyrene beads through the optical traps constructed by the laser, and then move the laser to apply the pulling force. Optical tweezers can achieve relatively high resolution (Abbondanzieri *et al*. 2005; Mahamdeh and Schäffer 2009), but it is difficult to keep constant force when tracking the conformational changes. Besides that, optical tweezers are not suitable for long time measurement, because high-intensity laser may damage the sample (Simpson *et al*. 1998). The magnetic tweezers connect the two ends of biological macromolecules to surface and paramagnetic bead respectively, and then apply the pulling force with an external magnetic field. Magnetic tweezers can track many biological macromolecules at the same time, but the resolution is slightly lower than optical tweezers. In this paper, we report a strategy to study the structural dynamics of chromatin fibers based on single-molecule magnetic tweezers. We modified the magnetic tweezers by adopting solid-microscope optical system, large-field objective lens, and high-speed sampling CCD camera to improve the time resolution of magnetic tweezers and the number of magnetic beads tracked at the same time. We also developed a home-built control software of magnetic tweezers based on LabVIEW to achieve more advanced bead tracking and data analysis algorithm, which further improves the resolution of magnetic beads position and pulling force. The chromatin fiber labeled with either biotin or digoxigenin at the two ends of DNA was reconstituted *in vitro* and attached to the modified magnetic tweezers. The extensions of chromatin fibers under different tension were recorded at single-molecule level to trace the dynamic structural transition. This strategy provides a new research platform to dissect the structural dynamics of chromatin fiber and its regulation by different chromatin factors.

## Experimental Section

### Preparation of chromatin fibers

Recombinant histones and DNA templates of 24 tandem 177 bp repeats of the 601 sequence (24 × 177 bp) were cloned and purified as previously described (Li *et al*. 2010). The 24 × 177 bp 601 DNA was subcloned into a modified pWM530 plasmid containing two BseYI sites flanking multiple cloning sites. The pWM530-BseYI-177-24 plasmid was digested by BseYI enzyme, with the 24 × 177 DNA templates purified by gel extraction. The two single-stranded ends of 24 × 177 DNA templates were filled with either dUTP-digoxigenin or dATP-biotin by Klenow reaction at 30 °C for 3 h. Remaining enzymes were removed by phenolic chloroform extraction and ethanol precipitation. For histone purification, pET-histone expression plasmids were transformed into BL21 (DE3) pLysS cells individually and then a single colony each was inoculated from a freshly streaked agar plate into 5 ml of LB medium containing 100 μg/mL ampicillin and 25 μg/mL chloramphenicol. 100 ml overnight culture from optimal clones was inoculated into 5 l LB medium and overexpression of histones were induced at* OD*_600 nm_ of 0.4–0.6 with 0.5 mmol/L IPTG. After growing for another 3–4 h, cells were harvested by centrifugation and resuspended with buffer A (50 mmol/L Tris–HCl, pH 7.5, 100 mmol/L NaCl, 1 mmol/L EDTA, 1 mmol/L bezamidine). After sonication and centrifugation, the pellets containing inclusion bodies of the corresponding histones were washed three times by buffer A plus 1% Triton X-100 and later washed with buffer A twice to get rid of Triton X-100. Then pellets were dissolved by gently stirring for 1 h at room temperature with buffer B (7 mol/L guanidinium HCl, 20 mmol/L Tris–HCl, pH 7.5, 10 mmol/L DTT). With another centrifugation (23,000 *g* for 10 min) to remove undissolved pellets, supernatants containing unfolded histones were collected and analyzed by 15% SDS-PAGE.

The respective histone octamers were reconstituted as previously described (Dyer *et al*. 2004). Equimolar amounts of individual histones in unfolding buffer B (7 mol/L guanidinium HCl, 20 mmol/L Tris–HCl, pH 7.5, 10 mmol/L DTT) were dialyzed into refolding buffer (2 mol/L NaCl, 10 mmol/L Tris–HCl, pH 7.5, 1 mmol/L EDTA, 5 mmol/L 2-mercaptoethanol), and purified through a Superdex S200 column. Chromatin samples were assembled using the salt-dialysis method as previously described (Song *et al*. 2014). The reconstitution reaction mixture with histone octamers and DNA templates in TEN buffers (10 mmol/L Tris–HCl, pH 8.0, 1 mmol/L EDTA, 2 mol/L NaCl) were dialyzed for 16 h at 4 °C in TEN buffer, which was continuously diluted by slowly pumping in TE buffer (10 mmol/L Tris–HCl, pH 8.0, 1 mmol/L EDTA) to a lower concentration of NaCl from 2 to 0.6 mol/L. For nucleosomal arrays, samples were collected after final dialysis in measurement HE buffer (10 mmol/L HEPES, pH 8.0, 0.1 mmol/L EDTA) for 4 h. For histone H1 incorporation, an equal molar amount of histone H1 (relative to mono-nucleosomes) was added before the final dialysis step and further dialyzed in TE buffer with 0.6 mol/L NaCl for 3 h. The stoichiometry of histone octamer binding to the DNA template was determined by EM investigation (Song *et al*. 2014).

Stoichiometry of histone octamer to DNA template and histone H1 to nucleosome were evaluated by EM analysis as described previously (Chen *et al*. 2013). The samples were fixed with 0.4% glutaraldehyde (Fluka) in HE buffer on ice for 30 min. For metal shadowing experiment, chromatin samples were prepared in HE buffer with DNA concentrations of 5 µg/mL. 2 mmol/L spermidine was added into the sample solution to enhance the absorption of chromatin to the grids. Samples were applied to the glow-discharged carbon-coated EM grids and incubated for 2 min and then blotted. Grids were washed stepwise in 20 ml baths of 0%, 25%, 50%, 75%, and 100% ethanol solution for 4 min, each at room temperature, air dried and then shadowed with tungsten at an angle of 10° with rotation. For negative staining studies, chromatin samples were prepared in HE buffer with DNA concentrations of 20 µg/mL. Chromatin samples in fixative solution were incubated on glow-discharged carbon-coated EM grids for 1 min. The excess sample solution was removed using filter papers. The grid was stained in 2% uranylacetate for 2 × 30 s, blotted with filter papers and allowed to air-dry for several minutes. Samples were examined using a FEI Tecnai G2 Spirit 120 kV transmission electron microscope.

### Setup of magnetic tweezers

**Fig. 1:**
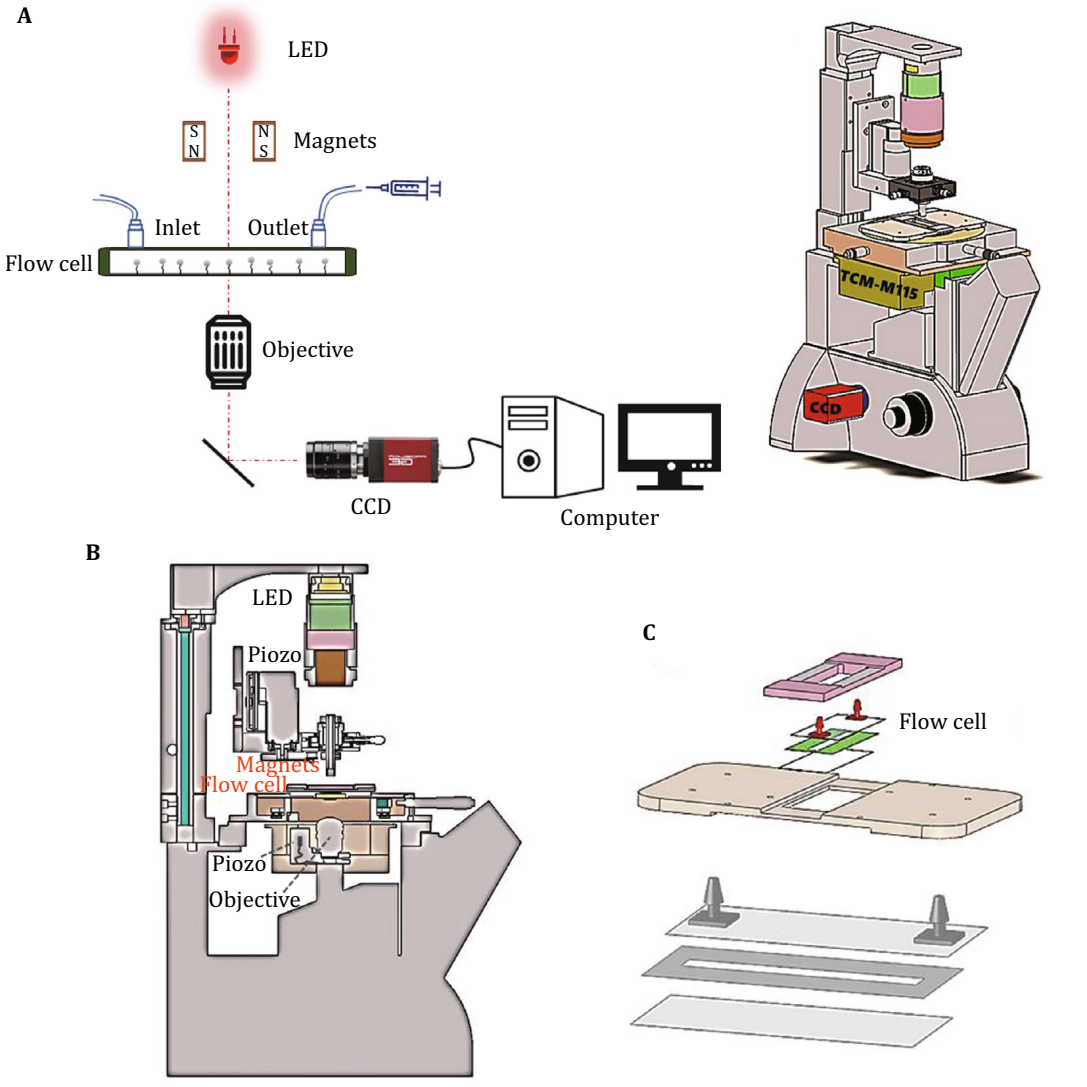
Setup of magnetic tweezers. **A** The schematic (left) and assembly (right) diagram of magnetic tweezers. **B** Sectional view of magnetic tweezers. Magnetic tweezers are consisted of LED light source, a pair of magnets, a flow cell, a flow cell and a CCD camera. **C** The configuration of the flow cell. The flow cell is formed by two coverslips with a two-sided tap in the center

As shown in Fig. 1, the setup of magnetic tweezers is mainly composed of light source (625 nm red LED, THORLABS), two NdFeB magnets with 0.5 mm, flow cell, 60× oil immersed objective (UPLSAPO60XO, NA 1.35, Olympus), and 1280 × 2024 CCD camera (MC1362, Mikrotron). The flow cell consists of a bottom coverslip, a double-sided tap with a rectangular channel (5 × 50 mm^2^), and an upper coverslip with an inlet and outer at each end. In the flow cell, one end of the chromatin fiber is anchored to the streptavidin coated magnetic beads (M280 Invitrogen Norway), and the other end is anchored to the surface coated with anti-digoxigenin. The two magnets are fixed on a high-resolution translation stage (M-126, PI) and a rotation stage (C-150, PI). The exerted tension (0–100 pN) on chromatin fibers is controlled by tuning the distance of the magnets in *z* direction (Dammer *et al*. 1996; Lee *et al*. 1994; Merkel *et al*. 1999; Moy *et al*. 1994; Pincet and Husson 2005). At a given magnet position, the tension exerted on the chromatin samples is constant. The twist can be regulated in the twist-constrained assays by rotating the magnets. The images of beads would go through the objective and then into the CCD camera. The thermal drift is the barrier for the image tracing in a long time up to several hours. In our magnetic tweezers, four Peltier modules are applied to control the sample temperature with the resolution of 0.1 °C.

### Coverslip cleaning and surface functionalization

To anchor the digoxin and biotin labeled chromatin fiber on the surface, the bottom coverslip needs to be cleaned and functionalized. Firstly, several pieces of coverslips were put in a staining jar filled with deionized water and detergent, and then the staining jar was placed in an ultrasonic cleaning bath for 30 min. After that, the coverslips were rinsed with deionized water for 5–10 times to remove the detergent. The washing process was repeated using acetone and methanol, respectively to rinse some organic impurities. To functionalize the surface of coverslip, the nitrocellulose solution (0.4%–0.8% w/v) was coated on the surface and heated at 120 °C for 3 min, then the surface could bind with anti-digoxin for tethering chromatin fibers (Cnossen *et al*. 2014).

To control the thermal drift, furthermore, the diluted polystyrene beads solution was dropped on the surface and heated at ~150 °C for 5 min to melt on the cover-slip surface. The fixed beads were tracked simultaneously and served as the indicator of the drift. We subtracted these drift signals in our LabVIEW software.

### Result

#### Preparation of chromatin fibers

**Fig. 2:**
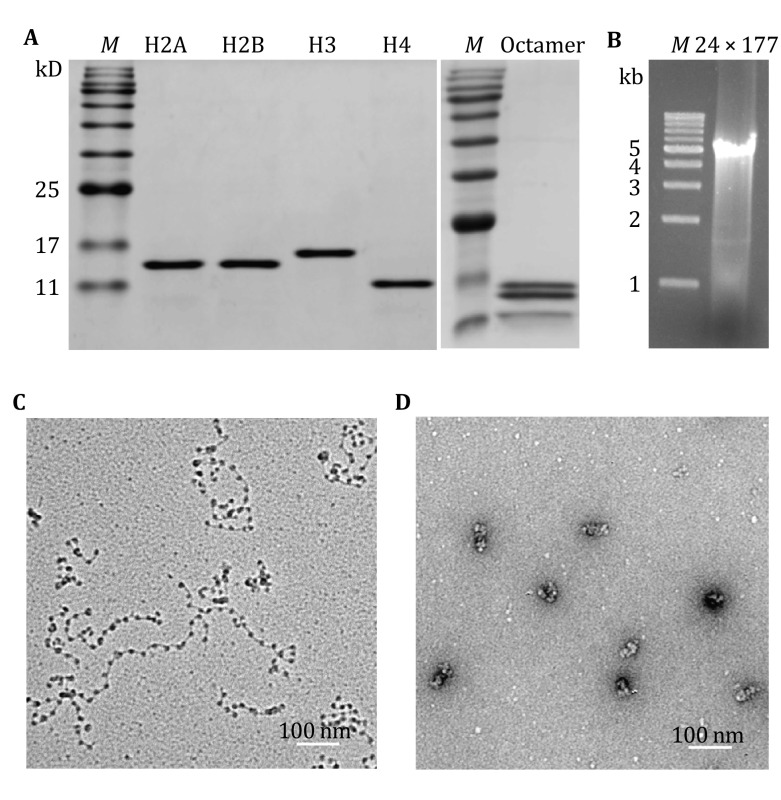
Preparation of chromatin samples. **A** SDS-PAGE analysis of the purified four histones and the reconstituted histone octamers. **B** Agarose gel analysis of the purified 24 × 177 bp DNA template for magnetic tweezers. **C** EM (metal shadowing) images of the nucleosomal arrays without H1. **D** EM (negatively stained) images of the compact chromatin fibers with H1

The nucleosomal arrays and chromatin fibers used for the single-molecule magnetic tweezers investigation were reconstituted *in vitro* by purified histones and DNA template (Fig. 2A, B). The DNA template containing 24 tandem repeats of 177-bp Widom 601 nucleosome positioning sequence (24 × 177 bp) was labeled with either biotin or digoxigenin at the two ends. In the absence of linker histone, the nucleosomal arrays reconstituted on the 24 × 177 bp DNA templates adopted an extended beads-on-a-string conformation, as shown by EM analysis (Fig. 2C). In the presence of H1, the arrays were further condensed into compact chromatin fibers (Fig. 2D).

The chromatin fiber labeled with either biotin or digoxigenin at the two ends was attached to the modified magnetic tweezers. To avoid non-specific binding of chromatin fibers to the surface and magnetic beads, the flow cell requires passivation by incubating with PBS passivation buffer (10 mg/mL BSA, 1 mmol/L EDTA, 10 mmol/L PB, 10 mg/mL Pluronic F127 surfactant (Sigma-Aldrich), 3 mmol/L NaN_3_, pH 7.8) for 2 h (Fulconis *et al*. 2006; Koster *et al*. 2005). To tether the chromatin fiber to the surface of super paramagnetic beads (M280 Invitrogen Norway), the diluted chromatin fiber solution (~10 pmol/L) was first mixed with M280 beads for 30 min at room temperature on the Hula Mixer (ThermoFisher) at 1 turns/min. Secondly, passivation buffer in the flow cell was rinsed by HE buffer (10 mmol/L HEPES, 1 mmol/L EDTA) using a pump (500 μl/min). Thirdly, the mixture of chromatin fibers and magnetic beads was incubated in the flow cell for 10 min to anchor the other end of the chromatin fiber to the surface of the functionalized coverslip. At last, the unbound beads and chromatin fibers in the flow cell were subsequently washed out with 500 μl HE buffer.

To identify the beads that only bind with single chromatin fiber, we pull the beads with a relatively large force (2–3 pN) and rotate the permanent magnets ~50 turns. If the bead binds more than one chromatin fiber, the chromatin fibers would be braided and the distance of the bead to surface would decrease. On the other hand, the beads that only bind single chromatin fiber have no changes in distance to surface, which could be tracked in further stretching experiments.

#### Tracking the magnetic beads in three-dimensions

In stretching experiments, the extension of chromatin fibers is determined by tracking the three-dimensional (3D) position of the magnetic beads. The positions of a magnetic bead in the horizontal direction (*X*, *Y*) can be determined by calculating the center of its diffraction pattern. A common bead tracking method is one-dimensional (1D) cross-correlation algorithm (Gosse and Croquette 2002), but the accuracy is relatively low. Our magnetic tweezers use the quadrant interpolation algorithm developed by Cees Dekker (van Loenhout *et al*. 2012), which is an improved version of conventional 1D cross-correlation algorithm. In brief, we calculate the estimated center (*X*_0_, *Y*_0_) of a bead using a center-of-mass computation, which is shown as follow:

1 $$X_{0} = \frac{ {\mathop \sum\limits_{i = 1}^{N} {\sum\limits_{j = 1}^{N} {(iI_{ij} )} } }}{{\sum\limits_{i = 1}^{N} {\sum\limits_{j = 1}^{N} {I_{ij} } } }},\quad Y_{0} = \frac{{\sum\limits_{i = 1}^{N} {\sum\limits_{j = 1}^{N} {(jI_{ij} )} } }}{{\sum\limits_{i = 1}^{N} {\sum\limits_{j = 1}^{N} {I_{ij} } } }},$$

where *i* and *j* are pixel subscripts of extracted bead image and *I*_*ij*_ represents the light intensity of the *j*th pixel point of the *i*th line. Based on the estimated center, the image is divided into four quadrants to sample radial profiles at polar coordinates, respectively. When calculating the *X* position of a bead, the sum of right radial profiles is concatenated with the left profiles, thus creating an intensity profile *I*_*x*_(*r*) along the *x* direction. *I*_*x*_(*r*) is cross-correlated with its mirror profile *I*_*x*_(−*r*):

2 $$C_{xx} = {\text{IFFT}}({\text{FFT}}(I_{x} (r))\,*\,\overline{{{\text{FFT}}(I_{x} ( - r))}} ),$$

where FFT is fast Fourier transform and IFFT is inverse fast Fourier transform, *C*_*xx*_ is resulting cross correlative curve. The *X* position of bead can be derived from the peak position *d*_*r*_ of resulting cross correlative curve *C*_*xx*_ as follow:

3 $$X = X_{0} + \frac{{2d_{{r}} }}{\pi },$$

where *X*_0_ is *X* coordinate of estimated center, *π* is circumference rate. By creating the intensity profile along the *Y* direction, we could calculate the *Y* position accordingly.

**Fig. 3:**
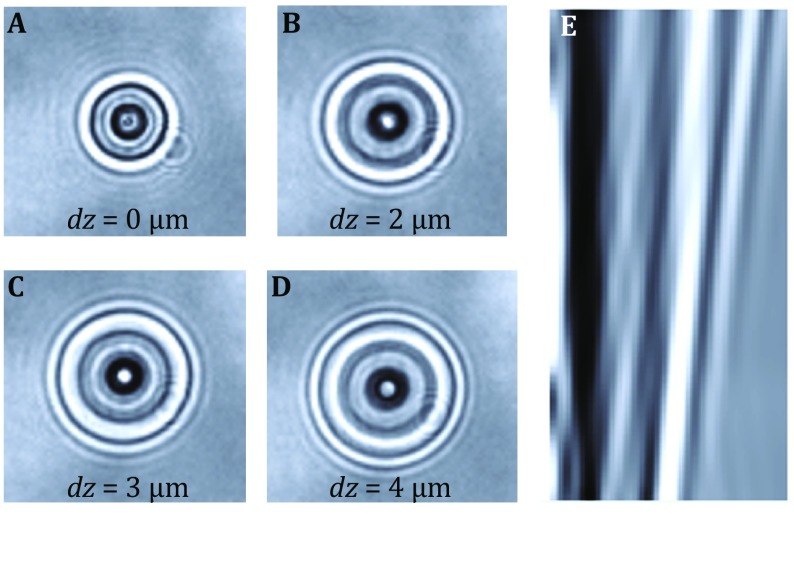
Generation of a calibration profile in the *z* direction for a bead. **A**–**D** The diffraction patterns of the bead with various distances to the focal plane. **E** By recording the size and density of the diffraction rings at different distance to the focal plane, a generated calibration profile is used to measure vertical relative displacements of the bead

Tracking the bead position in vertical direction (*z* direction) requires a prerequisite calibration that building a relationship between defocused distance and diffraction pattern of beads (Gosse and Croquette 2002). Firstly, by moving magnets toward flow cell, a relatively large force (~2–3 pN) is applied on beads to reduce the fluctuation of beads. Then the inversed objective is moving at ~100 nm step size, while the CCD is recording the diffraction pattern at every step (Fig. 3A–D). The relationship between diffraction patterns and defocused distance can create a two-dimensional (2D) look-up table (LUT) through bi-linear interpolation (Fig. 3E). To determine the *Z* position of a given image, the *χ*^2^ value of the radial profile *I*(*r*) with LUT is calculated:

4 $$\chi^{2} = \sum\limits_{r = 0}^{R} {(I(r) - I_{\text{LUT}} (r))^{2} } .$$

Thus, the minimum *χ*^2^ corresponds to the *Z* position of the image.

#### Calculation of applied force

**Fig. 4:**
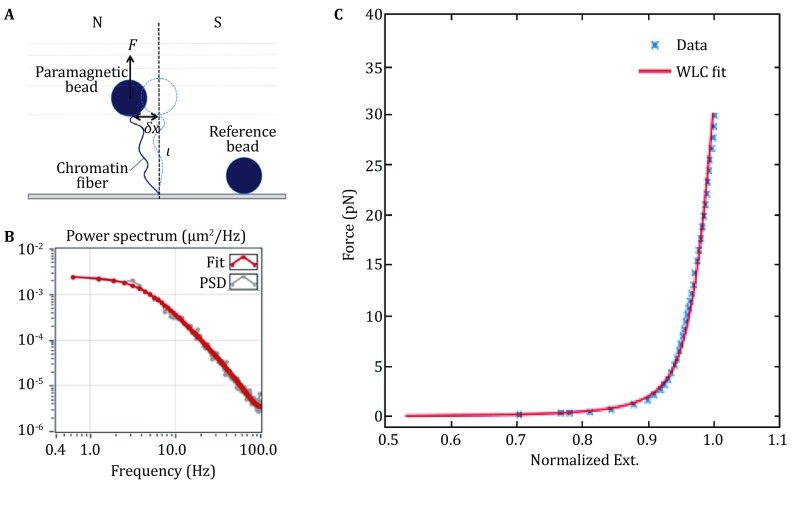
The force calculation for the DNA tether. **A** A schematic representation of a tethered bead with a reference bead fixed to the bottom coverslip. The exerted tension *F* on the paramagnetic bead arises from the gradient of the magnetic field. **B** The spectrum and the fitted curve of the one-dimensional fluctuations of the paramagnetic bead under a constant tension. **C** For a 10 kb dsDNA, the relationship between the measured force and extension is consistent with the WLC (worm-like chain) model

To calculate force applied on samples, a common used method is treating the bead–chromatin system as an inversed pendulum with small displacements in the vicinity equilibrium position (Klaue and Seidel 2009; Strick *et al*. 1998). In this model, the magnetic bead is attached to a rigid rod with length *l*, and the force *F*_mag_ exerts on it. When the pendulum has a small displacement *a*, restoring force is giving by $F_{a} = - \frac{{F_{\text{mag}} }}{l} \times a.$ This equation is equivalent to the rule of a Hookean spring with the spring constant $k_{a} = \frac{{F_{\text{mag}} }}{l},$ according the equipartition theorem,

5 $$E_{\text{therm}} = \frac{1}{2}k_{a} \langle a^{2} \rangle = \frac{1}{2}k_{\text{B}} T,$$

where *k*_B_ is Boltzmann constant and *T* is temperature. Then we get the force *F*_mag_:

6 $$F_{\text{mag}} = \frac{{k_{\text{B}} T}}{{\langle a^{2} \rangle }}l,$$

where $\langle a^{2} \rangle$ is the variance of bead in lateral fluctuation, and *l* is effective pendulum length, namely the sum of radius of bead and extension of chromatin fiber. Therefore, the applied force can be determined from the lateral fluctuation of the bead (Fig. 4A, B**)**. However, in practice, at forces greater than ~10 pN, the measurement of $\langle a^{2} \rangle$ in real space appears systematic error because of the finite camera acquisition frequency. Therefore, power spectral density (PSD) analysis is typically used to analyze the in-plane fluctuations.

Considering the viscous drag coefficient *γ*_*a*_, the PSD can be expressed:

7 $$S_{a} (f) = \frac{{4k_{\text{B}} T\gamma_{a} }}{{k_{a}^{2} }}\frac{1}{{1 + (f/f_{\text{c}} )^{2} }},$$

where *f* is the frequency and $f_{\text{c}} = \frac{{k_{a} }}{{2\pi \gamma_{a} }}$ is characteristic cutoff frequency. Due to the limiting of acquisition frequency of camera *f*_s_, low-pass filtering and aliasing should be considered (Klaue and Seidel 2009; Lansdorp and Saleh 2012; te Velthuis *et al*. 2010) as follows,

8 $$S_{a}^{\text{corr}} (f) = \sum\limits_{n = - \infty }^{\infty } {\frac{{4k_{\text{B}} T\gamma_{a} }}{{k_{a}^{2} }}} \frac{1}{{1 + (|f + nf_{\text{s}} |/f_{\text{c}} )^{2} }}\frac{{\sin^{2} (\pi \tau_{\text{e}} |f + nf_{\text{s}} |)}}{{(\pi \tau_{\text{e}} |f + nf_{\text{s}} |)^{2} }},$$

where *τ*_e_ is the exposure time of the camera. This formula can be used to fit the PSD, obtaining the *k*_*a*_, then the *F*_mag_. In our experiment, we used an improved PSD fitting method developed by Ralf Seidel, which takes into account the exposure time, direction of magnet field and rotation of bead (Daldrop *et al*. 2015).

To calibrate the force derived from our magnetic tweezers, we stretched a dsDNA with a length of 10 kb, then we derived the applied force from lateral fluctuation of beads and fitted with an extensible WLC model (Odijk 1995):

9 $$\frac{z}{L} = 1 - \frac{1}{2}\left( {\frac{{k_{\text{B}} T}}{{FL_{\text{P}} }}} \right)^{1/2} + \frac{F}{S},$$

where *z* and *L* are extension and the contour length of molecule, *L*_P_ is persistence length that characterize the rigidity of molecule, and *S* is stretch modulus of the molecule. As shown in Fig. 4C, our experiment result agrees well with the WLC model, indicating a good performance of our magnetic tweezers.

#### Structural dynamics of chromatin fibers revealed by magnetic tweezers

**Fig. 5:**
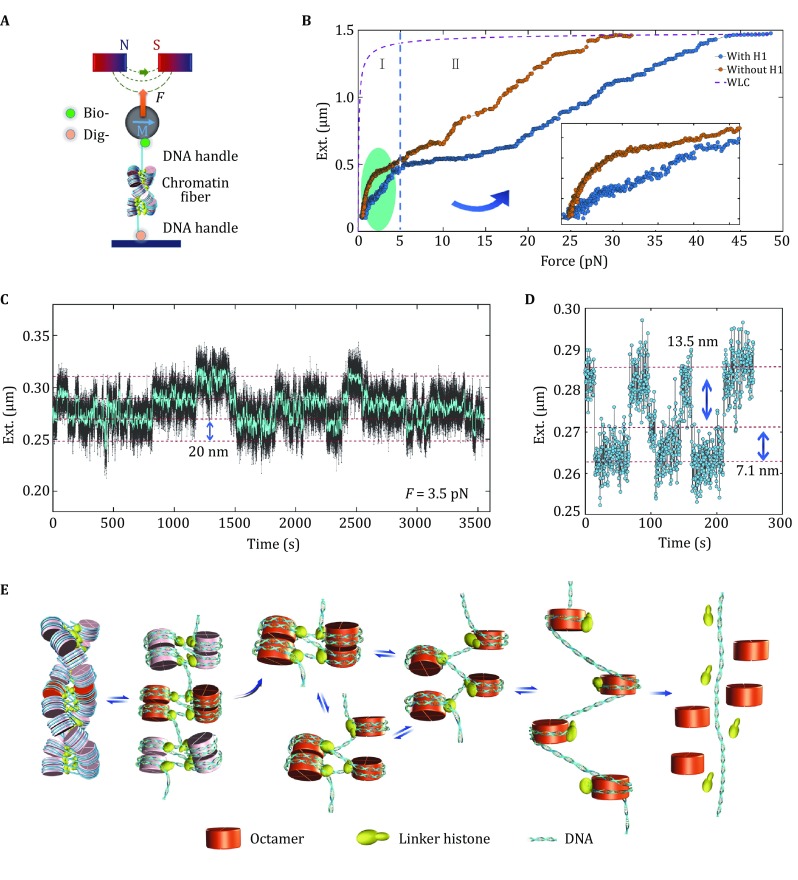
Investigation of structural dynamics of chromatin fibers by single-molecule magnetic tweezers. **A** Schematic representation of chromatin fiber studied by magnetic tweezers. **B** Comparison of two typical force–extension curves of a chromatin fiber with H1 (*blue curve*) and without H1 (*orange curve*) in HE buffer, with two major stages at high force (>5 pN) or low force (<5 pN) recognized. The inset shows the details of stage at low force (<5 pN). **C** Stepwise folding and unfolding dynamics of tetranucleosomal units at 3.5 pN for the chromatin fiber with H1. **D** Stepwise folding and unfolding dynamics of each tetranucleosomal unit with two alternative pathways at 3.5 pN for the chromatin fiber with H1. **E** Model for the dynamic organization of chromatin fiber. The left-handed double-helix chromatin fiber unfolds to a “tetranucleosomes-on-a string” extended structure, and then unfolds to a complete open nucleosomal array in one or two steps Adapted with permission, from Li *et al*. (2016a, b, c)

Using our improved magnetic tweezers, we investigated the hierarchical organization and structural dynamics of chromatin fibers reconstituted *in vitro* in the presence of H1 (Fig. 5A), whose 3D Cryo-EM structures have been resolved recently (Li *et al*. 2016c; Song *et al*. 2014). To trace the structural transition, we applied a continuously increasing force on a single chromatin fiber by moving the magnets in *z* direction at 2 μm/s. The force–extension curve of a chromatin fiber containing H1 is shown by the blue curve, with that of a nucleosomal array without H1 for comparison (Fig. 5B). At force above 5 pN, similar stepwise unfolding events were observed for both extended nucleosomal arrays and compacted chromatin fibers, which correspond to the rupture states of nucleosomes in the chromatin fiber (Bintu *et al*. 2012; Brower-Toland *et al*. 2002; Hall *et al*. 2009). At low-force region (<5 pN), the curve of the chromatin fiber begins to deviate from that of the nucleosomal array at ~1 pN, and a distinct force plateau spanning a range of ~100 nm near 3 pN is clearly identified (Fig. 5B). The structural transition near 3 pN should be attributed to the disruption of nucleosome–nucleosome interactions in the higher-order chromatins, as the nucleosomes remain intact at such low forces.

In order to gain more insights into the nature of structural transition near 3 pN, we performed the force-clamp measurements of chromatin fibers. Interestingly, a reversible folding and unfolding dynamical behavior was observed at 3.5 pN (Fig. 5C), with the obvious transition step size of ~20 nm. The details of the folding and unfolding dynamics of chromatin fiber at 3.5 pN indicate that the structure transition with step of 20 nm is always accomplished within two steps, which corresponds to the two-step disruption of tetra-nucleosome structural unit (Fig. 5D). Importantly, similar dynamic processes can be observed in the chromatin fibers assembled not only on regular tandem repeat of 601 DNA sequence but also on the scrambled (non-repetitive) DNA sequence, suggesting that the existence of tetra-nucleosome units is not dependent on DNA sequence. By using the modified single-molecule magnetic tweezers method, we show that the tetra-nucleosome unit exists as a stable structural intermediate of the 30-nm chromatin fiber and further unfolds to a more extended “beads-on-a-string” conformation by disrupting the nucleosome–nucleosome interactions within tetranucleosomal unit (Fig. 5E) (Li *et al*. 2016b).

## Discussion

In eukaryotic gene activation, the compact chromatin fibers must unfold for transcription and other DNA-related biological processes. Chromatin dynamics is critical to regulate the accessibility of transcription factors via dynamic transitions between the compact chromatin fiber and the more accessible nucleosomal array *in vivo* (Li *et al*. 2010; Li and Reinberg 2011). Therefore, it is of great importance to study how the dynamic organization of the chromatin fiber is regulated. However, there are various technical challenges to probe the detailed structure and the dynamics of chromatin fiber using traditional biochemical assays (Vilfan *et al*. 2009). For example, static conformations obtained by Cryo-EM structural study *in vitro* cannot provide much detailed information for dynamic processes. As a single-molecule method, magnetic tweezers is a powerful tool to study the dynamic organization of chromatin fibers by tracing the real-time folding and unfolding of single chromatin fiber (Bintu *et al*. 2012; Brower-Toland *et al*. 2002; Hall *et al*. 2009; Kruithof *et al*. 2009; Meng *et al*. 2015; Yan *et al*. 2007).

In this paper, we present a platform for dissecting dynamics of chromatin fibers using single-molecule magnetic tweezers. With more advanced tracking algorithms and higher resolution, we can observe the unfolding details of chromatin fibers and monitor the folding dynamics of chromatin fibers by careful tension adjustment. With magnetic tweezers, we have revealed that tetra-nucleosome unit exists as a stable structural intermediate during the formation of 30-nm chromatin fiber. These results are the complement of traditional biochemical experiments and high-resolution static imaging techniques such as EM, depicting the dynamics of single chromatin fiber, and providing a new perspective for understanding the dynamic organization of chromatin fibers and its regulation by chromatin factors.

Magnetic tweezers are widely used in the study of biological macromolecules because of their simple principle, low cost, high parallelism and single-molecule tracking. In addition to studying the mechanical properties of DNA and its higher-order structures, magnetic tweezers can also be used to study the folding kinetics of various proteins, such as actin (Yuan *et al*. 2017) and trans-membrane proteins (Min *et al*. 2015). The constant force measurement, which is easy to be realized by magnetic tweezers, is also suitable for studying the working mechanism of DNA-related enzymes, such as condensin (Eeftens *et al*. 2017), helicase (Li *et al*. 2016a; Seol *et al*. 2016), and polymerase (Maier *et al*. 2000).

In the last 20 years, the technology and analytical theory of magnetic tweezers have been continuously improved. According to the needs of experiments, a series of improvements on magnetic tweezers have been made. For example, Yan Jie’s group developed the transverse magnetic tweezers, which can greatly improve the force applied by the magnetic beads (Yan *et al*. 2004). They also developed a non-disturbing buffer exchange technology to replace the buffer environment in the measurement process (Le *et al*. 2015). In order to improve the resolution of magnetic tweezers, Ralf Seidel *et al*. used CMOS camera with ultra-high sampling rate and image processing technology base on GPU, improving the resolution of magnetic tweezers to angstrom level (Huhle *et al*. 2015). On the other hand, Terence R. Strick *et al*. used single-molecule FRET technology to combine with magnetic tweezers (Graves *et al*. 2015), which not only can improve the resolution of length change, but also can be used to reveal whether the protein falls off in the process of a structural transformation. Further development of these technologies may provide new platform to solve the hard questions in life science.
